# Moyamoya disease presenting with transient nonfocal neurological attacks in an Indian woman carrying a previously unreported RNF213 missense variant (p.Thr554Ile)

**DOI:** 10.1007/s10048-025-00836-5

**Published:** 2025-07-19

**Authors:** Ritwick Mondal, Shramana Deb, Nirmalya Ray, Sukalyan Purakayastha, Mona Tiwari, Julián Benito-León, Jayanta Roy

**Affiliations:** 1 Department of Stroke Medicine, Institute of Neurosciences, Kolkata, India; 2 Department of Neuroradiology, Manipal Group of Hospitals, Kolkata, India; 3 Department of Interventional Neurology, Institute of Neurosciences, Kolkata, India; 4 Department of Neuroradiology, Institute of Neurosciences, Kolkata, India; 5 Department of Neurology, 12 de Octubre University Hospital, Madrid, Spain; 6 Instituto de Investigación Sanitaria Hospital 12 de Octubre (imas12), Madrid, Spain; 7 Centro de Investigación Biomédica en Red Sobre Enfermedades Neurodegenerativas (CIBERNED), Madrid, Spain; 8 Department of Medicine, Complutense University, Madrid, Spain

**Keywords:** Moyamoya disease, Transient neurological attack, Transient ischemic attack, Supraclinoid internal carotid artery stenosis, Digital Subtraction angiography, RNF213, Thr554Ile variant, Revascularization, Superficial temporal artery to middle cerebral artery bypass, Genetic susceptibility, Adult-onset moyamoya disease

## Abstract

Moyamoya disease is a rare cerebrovascular disorder characterized by progressive internal carotid artery stenosis and compensatory collateral vessel formation, producing a characteristic “puff of smoke” angiographic appearance. Genetic predisposition, particularly involving the RNF213 gene, plays a central role. We report a 48-year‐old Indian woman with type 2 diabetes, arterial hypertension, and a prior transient ischemic attack who presented with intermittent bilateral upper limb paresthesia. Imaging revealed bilateral supraclinoid internal carotid artery stenosis (Suzuki stage III). Genetic testing identified a heterozygous RNF213 missense variant (Thr554Ile, rs766831703), which is extremely rare in global databases and predicted to be deleterious by multiple in silico tools. This variant has not been previously described in association with Moyamoya disease. The patient underwent bilateral superficial temporal artery to middle cerebral artery bypass, achieving sustained clinical improvement without recurrent events over two years.

## Background

Moyamoya disease is an idiopathic, sometimes familial cerebrovascular disorder characterized by progressive steno-occlusive changes affecting the terminal internal carotid artery (ICA) and circle of Willis (the “carotid fork”). The compensatory collateral vessels produce the classic “puff of smoke” appearance on angiography [1].

The RNF213 gene, located on chromosome 17q25.4, is a key susceptibility gene for moyamoya disease, with multiple single nucleotide polymorphisms (SNPs) identified. Among these, the p.R4810K mutation is strongly associated with moyamoya disease in East Asians, with odds ratios of 184.04, 109.77, and 31.53 in Japan, Korea, and China, respectively [2]. However, the role of non-p. R4810K variants remains unclear.

De novo variants in adult-onset moyamoya disease are exceptionally rare, with only a few reported cases worldwide [3]. This report describes the clinical course of an adult female with a previously unreported RNF213 mutation, expanding the spectrum of genetic variants associated with the disease.

## Case presentation

A 48-year-old Indian woman with type 2 diabetes mellitus, arterial hypertension, and primary hypothyroidism presented with intermittent bilateral upper limb paresthesia that had persisted for a year and worsened over the two days before admission. She had no history of falls, seizures, loss of consciousness, vomiting, trauma, or bowel and bladder incontinence. She had been on thyroxine supplementation for five years. A detailed history revealed a prior right hemispheric transient ischemic attack (TIA), characterized by slurred speech and right-sided facial weakness, which resolved spontaneously in less than an hour. At that time, she had received antithrombotic and statin therapy, but no comprehensive neurovascular evaluation was performed. Following this episode, she developed intermittent episodes of paraesthesia and tingling sensation of variable duration, lasting from a few seconds to minutes in both upper limbs, for which she was prescribed gabapentin and pregabalin on an as-needed basis. Over time, she experienced sleep disturbances and acute anxiety episodes, but she remained functionally independent until her symptoms worsened significantly.

Her medical history included a cholecystectomy six years earlier and no history of substance abuse or intoxication. A neurological examination did not reveal any objective sensory or motor deficits. A comprehensive biochemical evaluation was unremarkable (Table 1). She was admitted to the stroke unit for further assessment and initiated on aspirin (75 mg twice daily), clopidogrel (75 mg once daily), and rosuvastatin (80 mg once daily), in addition to her existing antihyperglycemic and thyroxine therapy.

Neuroimaging studies began with non-contrast computed tomography and magnetic resonance imaging of the brain and spine, which were unremarkable. The nerve conduction study of the bilateral upper limbs was also unremarkable. Based on these findings, a provisional diagnosis of nonfocal transient neurological attacks (TNAs) was considered. The differential diagnosis of TNAs is wide, ranging from a demyelinating disease (multiple sclerosis), an entrapment neuropathy, cervical radiculopathy, or rarely a sensory seizure phenomenon. As the TNAs are also a warning sign of underlying cerebrovascular disorders, subsequent magnetic resonance angiography with time-of-flight was performed, which revealed bilateral supraclinoid internal carotid artery (ICA) stenosis (right greater than left). A six-vessel digital subtraction angiography (DSA) was performed to characterize these findings further.

**Fig. 1:**
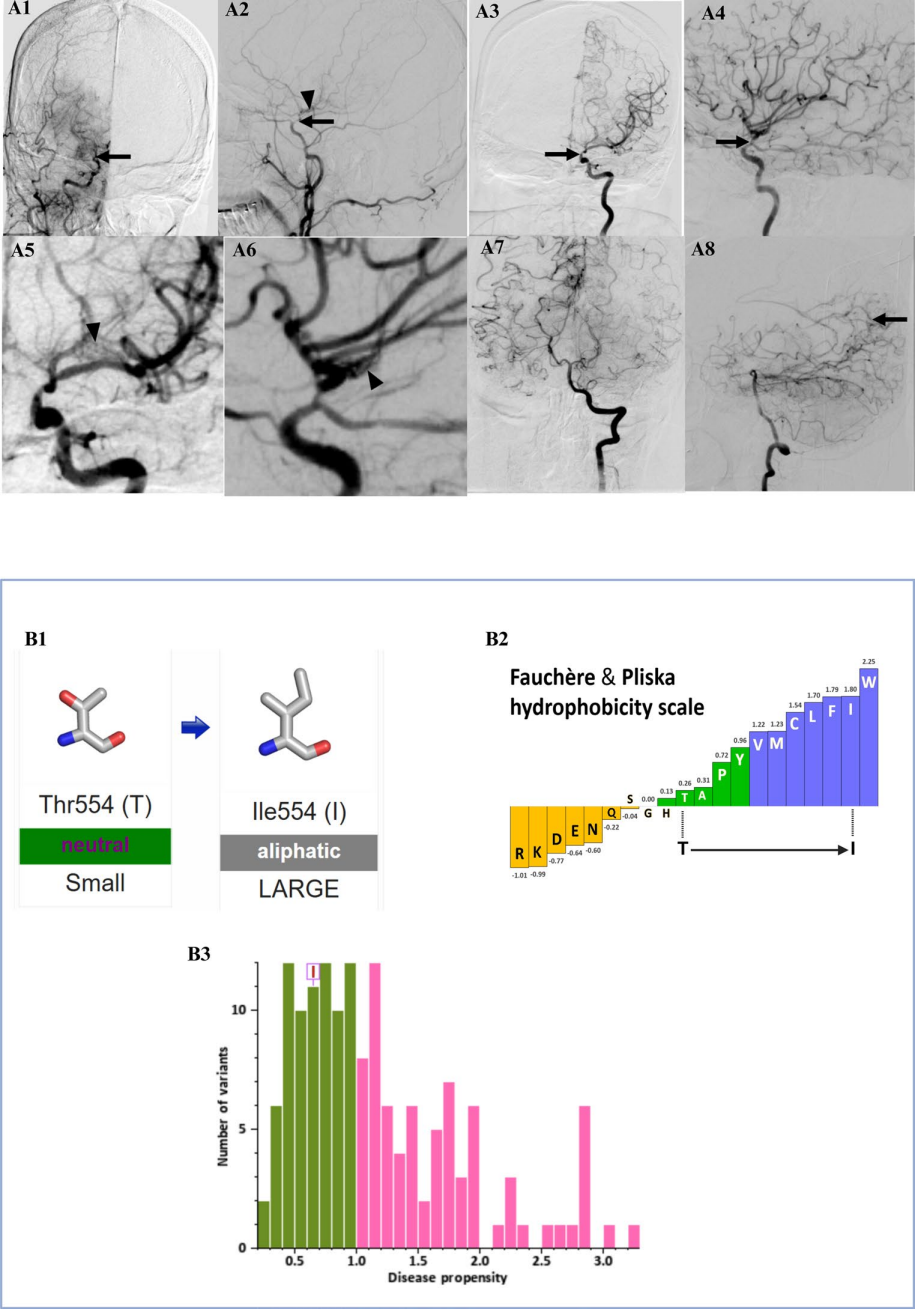
**(A1)** **and (A2):** Right internal carotid artery (ICA) angiograms in anteroposterior and lateral projections show abrupt supraclinoid narrowing (black arrows). A few lenticulostriate collaterals are noted in the right capsuloganglionic region (arrowhead in A2). There is a marked reduction in opacification of the right anterior and middle cerebral arteries and their branches. **(A3) and (A4):** Left ICA injections in anteroposterior and lateral projections reveal moderate narrowing of the supraclinoid segment (black arrows), with better preserved distal filling. **(A5)** and **(A6):** Magnified views of the left ICA show numerous lenticulostriate collaterals forming a characteristic “puff-of-smoke” appearance in the left capsuloganglionic region (arrowheads). **(A7) and (A8):** Left vertebral artery angiograms in anteroposterior and lateral views show partial reconstitution of the right anterior and middle cerebral artery branches through posterior circulation. Pial–pial collaterals between anterior and posterior cerebral arteries are seen (arrow in A8). **(B1):** Amino acid substitution at residue 554 in the RNF213 protein: threonine (Thr), a polar uncharged residue, is replaced by isoleucine (Ile), a larger hydrophobic aliphatic residue. This physicochemical change may affect local protein structure or function. **(B2):** Hydrophobicity plot based on the Fauchère and Pliska scale. The position of the Thr554Ile variant is clearly indicated with an enhanced arrow marker, demonstrating an increase in hydrophobicity from threonine to isoleucine. **(B3):** Histogram of disease propensity scores for RNF213 variants. The Thr554Ile substitution is highlighted (boxed), with a disease propensity value of 0.66. Scores are derived from VarSite and gnomAD databases, reflecting the frequency of each variant in disease versus natural populations

DSA demonstrated an abrupt narrowing of the right supraclinoid ICA with lenticulostriate collaterals in the right capsuloganglionic region. A significant reduction in flow was observed in the right anterior and middle cerebral arteries and their branches. Moderate narrowing of the left supraclinoid ICA was also noted, along with multiple lenticulostriate collaterals, forming the characteristic “puff of smoke” appearance in the left capsuloganglionic region. Partial reformation of the right anterior and middle cerebral artery branches from the posterior circulation was seen, along with pial-pial collaterals between anterior and posterior cerebral artery branches (Fig. 1). These findings supported a provisional diagnosis of bilateral moyamoya disease (Suzuki stage III), prompting a clinical exome sequencing panel of moyamoya disease for genetic confirmation.

### Genetic analysis

Following the patient’s consent and according to our institutional protocol, a clinical exome sequencing panel for moyamoya disease was subsequently performed. Genomic DNA was extracted from peripheral blood using a Qiagen isolation kit. Library preparation followed the SureSelect XTHS2 protocol, and sequencing was performed on an Illumina NovaSeq 6000 platform. Demultiplexing generated FastQ files, which underwent quality control using FastQC v0.12.1. High-quality reads were aligned to the hg38 reference genome (GRCh38) using BWA-MEM. Variant calling, including single nucleotide variants (SNVs) and small insertions/deletions (InDels), was performed using GATK-v4.3 and the SMART-One™ Tech Platform, following best-practice workflows.

The next genome sequencing analysis identified a heterozygous missense variant (c.1661 C > T) in the RNF213 gene at chromosome 17:80294909:C > T, located in exon 9 of transcript NM_001256071. This corresponds to the Thr554Ile variant in isoform 3 of the RNF213 protein (Q63HN8-3) (https://www.ncbi.nlm.nih.gov/protein/NP_001243000.2). This variant is cataloged as rs766831703 in the dbSNP database but has not been reported in ClinVar.

The variant was classified as benign by multiple in silico prediction tools (REVEL, CADD-PHRED, MutationTaster, and MetaLR). However, such tools are not definitive, and their reliability may be limited in genes with complex structure-function relationships such as RNF213 [4]. VarSite analysis suggested that substituting threonine with isoleucine (Ile) at position 554 could alter protein function, given its high conservation score (0.9 from 43 aligned sequences). Although the variant is located outside the canonical p.R4810K hotspot, RNF213 encodes a large multidomain protein with regions whose functional roles remain incompletely understood. The variant had no known disease-associated mutations and exhibited a moderate disease propensity score (0.66; Fig.1). Therefore, while current in silico predictors classify it as benign, the potential for subtle effects on protein structure or function—particularly in conserved, non-hotspot residues—justifies further functional investigation.

Due to financial constraints, family genetic screening was not performed. However, no first- or second-degree relatives had a history of cerebrovascular disease, including early-onset strokes, suggesting a de novo mutation as a likely pathogenic mechanism.

### Management and outcome

Given the high risk of stroke recurrence, the patient was advised to undergo a bilateral superficial temporal artery to middle cerebral artery (STA-MCA) bypass. The surgery was performed sequentially on both sides, with unremarkable perioperative outcomes. Over two years of follow-up, the patient remained stroke-free and neurologically stable, with no further ischemic events and preserved cognitive function (Table 1).

**Table 1:** Baseline and follow-up clinical, laboratory, and genetic findings

Parameters	Baseline Findings	Post-Surgical Follow-Up (up to 24 Months)
Vascular Risk Factors	Type 2 diabetes mellitus, arterial hypertension, and hypothyroidism	Maintained on medication
Neurological Presentation	- Tingling sensation in both upper limbs (worsened over the past two days) - Decreased fine touch sensation in both upper limbs - Normal speech - Muscle strength: 5/5 in all four limbs - Pupils: 2 + bilaterally - Decreased bilateral plantar reflexes - Glasgow Coma Scale: 15/15 - National Institutes of Health Stroke Scale (NIHSS): 1 - Modified Rankin Scale (mRS): 3 - Mini-Mental State Examination: 27/30 - Montreal Cognitive Assessment: 28/30	- NIHSS: 0 - mRS: 1 - Overall satisfactory neurological improvement
Stroke Presentation	Transient ischemic attack with a prior episode two years earlier	No recurrence post-surgery
Biochemical and Laboratory Findings	- Bleeding time: 2.35 s - Clotting time: 5.15 s - Prothrombin time: 13.2 s - Activated partial thromboplastin time (aPTT): 24.5 s - International normalized ratio (INR): 1.10 - Platelet count: 2.10 × 10⁵/µL - Erythrocyte sedimentation rate: 2 mm/hr - Serum sodium: 140 mEq/L - Serum potassium: 3.9 mEq/L - Serum creatinine: 0.75 mg/dL - Thrombophilia screening: Normal - Cerebrospinal fluid and serum autoimmune panel: Negative - Varicella zoster virus polymerase chain reaction: Negative - Sickle cell screening: Negative - Electroencephalography: Unremarkable	All parameters remained within the normal range.
Genetic Findings	- Gene and Transcript: RNF213 (NM_001256071) - Genomic Position: Chr 17:80294909 - Nucleotide Change: c.1661 C > T - Protein Change: p.Thr554Ile - rsID: rs766831703	

## Discussion

We describe an adult-onset case of moyamoya disease with nonfocal TNAs and a prior TIA associated with a rare RNF213 missense variant not previously linked to the disease. Given the absence of cerebrovascular disease in first- and second-degree relatives, this variant is strongly suspected to be a de novo pathogenic mutation leading to bilateral disease involvement in this middle-aged female.

Traditionally, moyamoya disease is considered a progressive childhood onset disorder, with classic angiographic features developing early in life. However, increasing evidence suggests that adult-onset moyamoya disease with significant disease progression is not as rare as previously thought [5]. Diagnosing moyamoya disease or moyamoya angiopathy in adults remains a challenge, particularly in patients presenting with subacute neurological symptoms in the form of nonfocal TNA, such as sensory disturbances, as seen in this case.

In this case, the patient presented with intermittent bilateral upper limb paresthesia—a symptom that, while uncommon, can be pathophysiologically explained by hemodynamic insufficiency in the setting of bilateral anterior circulation disease [6, 7]. The supraclinoid internal carotid arteries supply the middle cerebral arteries, which in turn perfuse the lateral sensorimotor cortex responsible for the upper limbs [7]. In moyamoya disease, insufficient collateralization and impaired cerebral autoregulation may result in transient bilateral neurological deficits, including sensory symptoms [8].

While disease progression is generally slower in adults than in children, approximately 20% of cases exhibit progressive steno-occlusive changes, regardless of initial symptomatology [9]. Moreover, the risk of recurrent stroke in these patients can reach 10% per year. Several studies have also identified female sex as an additional risk factor for disease progression [10].

Importantly, collateral vessel formation significantly influences clinical severity, recurrence risk, and long-term prognosis in ischemic moyamoya disease [11]. Literature suggests that not all cases of adult-onset moyamoya disease follow a progressive course; however, revascularization surgery remains pivotal in enhancing cerebral perfusion and lowering the risk of stroke recurrence [12]. Given that our patient was at high risk for disease progression due to both vascular risk factors and bilateral involvement, a bilateral direct STA-MCA bypass was performed rather than conservative antiplatelet therapy in alignment with the AHA/ASA 2021 guidelines and ESO Guidelines [13, 14]. Over 24 months of follow-up, she remained stroke-free and asymptomatic and maintained preserved cognitive function with a mRS of 1.

## Conclusion

Adult patients exhibiting subacute neurological or neurocognitive symptoms indicative of nonfocal TNAs, in combination with focal TNAs or TIAs—especially women with vascular risk factors—should undergo comprehensive angiographic assessment to exclude moyamoya disease or moyamoya angiopathy. In cases where involvement of the carotid fork is observed, genetic testing should be considered to detect potential pathogenic variants. Early diagnosis and timely revascularization can improve long-term outcomes and reduce stroke risk in adult-onset moyamoya disease. Further research is warranted to clarify the role of RNF213 polymorphisms in disease progression and their impact on adult-onset moyamoya disease phenotypes. The identification and reporting of novel RNF213 variants in adults with adult-onset moyamoya disease are critical to advancing our understanding of the disease’s heterogeneous manifestations. Such efforts can support the development of precision medicine approaches and contribute to expanding the genetic databases essential for optimizing patient care.

## Data Availability

No datasets were generated or analysed during the current study.
